# Gut Microbiota Mediate Insecticide Resistance in the Diamondback Moth, *Plutella xylostella* (L.)

**DOI:** 10.3389/fmicb.2018.00025

**Published:** 2018-01-23

**Authors:** Xiaofeng Xia, Botong Sun, Geoff M. Gurr, Liette Vasseur, Minqian Xue, Minsheng You

**Affiliations:** ^1^State Key Laboratory of Ecological Pest Control for Fujian and Taiwan Crops, Institute of Applied Ecology, Fujian Agriculture and Forestry University, Fuzhou, China; ^2^Joint International Research Laboratory of Ecological Pest Control, Ministry of Education, Fuzhou, China; ^3^Key Laboratory of Green Pest Control (Fujian Agriculture and Forestry University), Fujian Province University, Fuzhou, China; ^4^Fujian-Taiwan Joint Centre for Ecological Control of Crop Pests, Fujian Agriculture and Forestry University, Fuzhou, China; ^5^Graham Centre, Charles Sturt University, Orange, NSW, Australia; ^6^Department of Biological Sciences, Brock University, Ontario, ON, Canada

**Keywords:** diamondback moth, microbial symbionts, immunity, pleiotropic effects, gut bacteria

## Abstract

The development of insecticide resistance in insect pests is a worldwide concern and elucidating the underlying mechanisms is critical for effective crop protection. Recent studies have indicated potential links between insect gut microbiota and insecticide resistance and these may apply to the diamondback moth, *Plutella xylostella* (L.), a globally and economically important pest of cruciferous crops. We isolated *Enterococcus* sp. (Firmicutes), *Enterobacter* sp. (Proteobacteria), and *Serratia* sp. (Proteobacteria) from the guts of *P. xylostella* and analyzed the effects on, and underlying mechanisms of insecticide resistance. *Enterococcus* sp. enhanced resistance to the widely used insecticide, chlorpyrifos, in *P. xylostella*, while in contrast, *Serratia* sp. decreased resistance and *Enterobacter* sp. and all strains of heat-killed bacteria had no effect. Importantly, the direct degradation of chlorpyrifos *in vitro* was consistent among the three strains of bacteria. We found that *Enterococcus* sp., vitamin C, and acetylsalicylic acid enhanced insecticide resistance in *P. xylostella* and had similar effects on expression of *P. xylostella* antimicrobial peptides. Expression of cecropin was down-regulated by the two compounds, while gloverin was up-regulated. Bacteria that were not associated with insecticide resistance induced contrasting gene expression profiles to *Enterococcus* sp. and the compounds. Our studies confirmed that gut bacteria play an important role in *P. xylostella* insecticide resistance, but the main mechanism is not direct detoxification of insecticides by gut bacteria. We also suggest that the influence of gut bacteria on insecticide resistance may depend on effects on the immune system. Our work advances understanding of the evolution of insecticide resistance in this key pest and highlights directions for research into insecticide resistance in other insect pest species.

## Introduction

The animal gut is a complicated ecosystem inhabited by a large number of microbes that play important roles in insect physiology and behavior, such as food digestion (Warnecke et al., 2007), host nutrition (Engel et al., 2012), immune response (Ryu et al., 2010), pathogen defense (Dillon et al., 2005), plant specialization (McLean et al., 2011), and mating preference (Sharon et al., 2010). It is known that the insect gut micro-environment influences or may even determine the structure of the gut microbial community and the structure and diversity of the gut microbiota, together with their metabolic activities, may have physiological effects in insects (Zilber-Rosenberg and Rosenberg, 2008; Tang et al., 2012). Indeed, insect gut microbiota are considered to constitute an important organ in insects, however changes in environmental conditions are known to impact the symbiotic relationships between the organism and its microbiota and associated gene expression (Zilber-Rosenberg and Rosenberg, 2008; Possemiers et al., 2011). Recently, studies have increasingly suggested links between insect gut microbiota and insecticide resistance (Broderick et al., 2006; Kikuchi et al., 2012; Engel and Moran, 2013; Xia et al., 2013).

With growing concerns about the rapid rise in insecticide resistance in pests, there is a need to gain a mechanistic understanding of the roles that insect gut microbiota may have in the development of resistance. Some studies have explored the functions of the insect gut microbial communities and how they may contribute to insecticide resistance. Kikuchi et al. (2012), for example, demonstrated that the gut symbiont *Burkholderia* mediates insecticide resistance in *Riptortus pedestris* (Hemiptera) and insecticide resistance facilitated by the fenitrothion-degrading *Burkholderia* strains may be horizontally transferred to other insects (Kikuchi et al., 2012; Kikuchi and Yumoto, 2013). In their study of a different symbiont, Cheng et al. (2017) reported that trichlorphon-degrading strains of *Citrobacter* sp. (CF-BD) isolated from the gut of *Bactrocera dorsalis* (Diptera) increased insecticide resistance.

Conflicting roles of gut bacteria in resistance to the biological toxins of *Bacillus thuringiensis* (Bt) have been reported. In one study, an increase in midgut microbiota load of *Spodoptera exigua* (Lepidoptera) induced an increased tolerance to Bt, indicating a relationship between the gut bacteria and insecticide resistance (Hernández-Martínez et al., 2010), while, another study reported that Bt was ineffective against *Lymantria dispar* (Lepidoptera) when the gut bacteria had been treated with antibiotics, but following subsequent reestablishment of normal gut microbiota, including *Enterobacter* sp. lethality of Bt was restored (Broderick et al., 2006), whereas Frankenhuyzen et al. (2010) found that gut bacteria did not contribute to mortality in Bt treated *L. dispar*. Although gut microbiota-dependent mortality is known from other Lepidoptera, such as *Vanessa cardui, Manduca sexta*, and *Pieris rapae* (Broderick et al., 2009), there is evidence of inconsistencies in the activity of microbiota on Bt toxins within species. (Johnston and Crickmore, 2009) reported that although continuous exposure of *M. sexta* to antibiotics reduced pathogenicity of Bt, gut bacteria did not facilitate the activity of Bt toxins DiPel and Cry1Ac, and work by Raymond et al. (2009) indicated that the Bt toxin was effective in killing *Plutella xylostella* larvae that have been reared aseptically.

Although much work has been done on insecticide resistance mediated by gut microbiota, little is known about the underlying mechanisms. Two key processes have been suggested as drivers of development of resistance in insects: direct biodegradation of the pesticides by gut microbiota, such as *R. pedestris* (Kikuchi et al., 2012) and *B. dorsalis* (Cheng et al., 2017), and immune modulation, whereby development of an innate immune response is induced by microbiota (Broderick et al., 2010; Hernández-Martínez et al., 2010).

*Plutella xylostella* is a globally and economically important insect pest species that attacks cruciferous crops and has been found to be resistant to several classes of insecticide (Talekar and Shelton, 1993; Zalucki et al., 2012). We have previously examined the diversity of gut microbiota in *P. xylostella*, based on 16S rRNA sequencing, and found Proteobacteria, followed by Firmicutes, to be the most abundant bacterial phyla (Xia et al., 2013), accounting for 97% of the *P. xylostella* gut bacteria. It was found that insecticide-resistant strains of *P. xylostella* hosted more Firmicutes and fewer Proteobacteria than susceptible strains, where the proportion of gut Firmicutes increased with exposure to insecticide (Xia et al., 2013). These results indicated an association between *P. xylostella* gut microbiota and insecticide resistance, but a causal role of bacteria in conferring resistance rather than responding to insecticide exposure was not confirmed.

Vilanova et al. (2016) suggested that *Enterococcus* sp. isolated from *Hyles euphorbiae* confers tolerance to toxic natural latex and plant extracts and both *Serratia* sp. (Xu et al., 2007) and *Enterobacter* sp. (Singh et al., 2004), which are the common genera in the *P. xylostella* gut (Xia et al., 2017), have been associated with degradation of the insecticide, chlorpyrifos. Building on previous findings, this study aimed to explore the effects and mechanisms of gut microbiota in triggering insecticide resistance in *P. xylostella* by analyzing the direct biodegradation of chlorpyrifos *in vitro*, and assessing gut bacteria mediated immune modulation that contributes to the insecticide resistance.

## Materials and methods

### Insect rearing

*Plutella xylostella* used in this study were previously the focus of a genomics analysis (You et al., 2013). The colony was established in 2004 and individuals have been continuously reared on radish seedlings using the method described by Xia et al. (2015).

### Bacterial cultures

*Enterobacter* sp. (JQ396388), *Serratia* sp. (JQ396393), and *Enterococcus* sp. (KC150018), which were previously isolated from *P. xylostella* guts were cultured in LB medium (see Xia et al., 2015). The bacteria were incubated on a rotary shaker at 150 rpm at 37°C overnight in 100 mL of LB medium. The cultures were subsequently centrifuged and the LB medium was removed. The bacteria were washed with double distilled (dd) H_2_O to remove residues of the culture medium, and then diluted with ddH2O to a concentration of OD_600_ = 1.0. In order to study the effects of heat treatment on *P. xylostella* gut bacteria, the three bacterial strains, at densities of OD_600_ = 1.0, were heat killed at 70°C for 15 min and then 10 μL of the heat killed bacteria solution was spotted onto LB medium and cultured at 37°C for 24 h. Each experiment was replicated three times.

### *In vitro* degradation of chlorpyrifos

Content of chlorpyrifos in solution can be determined by ultraviolet (UV) spectrophotometry (Xie et al., 2005), so we constructed a standard curve by dissolving 25 mL of chlorpyrifos (100 mg L^−1^) in petroleum ether. The chlorpyrifos solution was diluted to concentrations of 0.0, 3.125, 6.25, 12.5, 25, 50, and 100 mg L^−1^ using petroleum ether, for measurement using a UV spectrophotometer at 293 nm absorbance. The standard curve, obtained using linear regression, was used to determine the concentration of degraded chlorpyrifos by the three bacterial strains.

To determine the degradation efficiency of the isolated strains of *Enterobacter* sp., *Serratia* sp., and *Enterococcus* sp., we first enriched the strains in LB liquid medium. When OD_600_ nm was about 0.5, the cells were harvested by centrifugation at 5,000 r min^−1^ for 5 min, before being washed twice in minimal salt (MS) solution (1.5 g K_2_HPO_4_, 0.5 g KH_2_PO_4_, 0.5 g (NH_4_)_2_SO_4_, 0.5 g NaCl, 0.2 g MgSO_4_, 0.05 g CaCl_2_, 0.02 g FeSO_4_, and 20 g agar in 1 L of ddH_2_O; pH 7.0) and re-suspended in MS solution to OD_600_ = 1.0. The bacteria (2% MS solution) were inoculated into 100 mL of MS medium that contained 50 mg L^−1^ of chlorpyrifos; the control containing an equal quantity of chlorpyrifos was not inoculated with bacteria. The control and bacteria treated insecticide MS media were incubated on the rotary shaker at 200 rpm at 37°C. Following incubation for 24 h, 4 mL of the culture medium was removed and an equal volume of petroleum ether was added before vortexing the mixture for 1 min. The organic and aqueous phases separated completely after 1 h, at which point the chlorpyrifos were extracted for analysis, where the organic phase solution of the upper layer was used to determine concentration at 293 nm absorbance. All experiments were replicated three times.

The degradation efficiency of chlorpyrifos was calculated as:

(1) $$X=({\text{C}}_{\text{CK}}-{\text{C}}_{\text{b}})/{\text{C}}_{\text{CK}}x100.$$

where *X* represented percent degradation efficiency; C_b_ was the concentration of chlorpyrifos in the medium degraded by a specific strain of bacteria (_b_) after culturing for 24 h; and, C_CK_ was the concentration of chlorpyrifos in the control group (no bacteria) that after culturing for 24 h under the same conditions. The degradation efficiency of pesticide was analyzed using one-way ANOVA, followed by an LSD *post-hoc* test.

### Colonization of *P. xylostella* gut bacteria

In order to detect colonization by the three strains of bacteria in the *P. xylostella* gut, cabbage leaves were dipped in a bacterial suspension of *Enterobacter* sp., *Serratia* sp. or *Enterococcus* sp. at OD_600_ = 1.0 for 10 min, and then air dried in Petri dishes. Cabbage leaves dipped in ddH_2_O and air dried as before were used as a control. There were three replicates of each treatment. Fifty first instar larvae were placed in a Petri dish containing cabbage leaves, until they reached the third instar. Control and treated cabbage leaves were renewed daily.

Quantitative PCR (qPCR) detection of gut bacteria was analyzed from randomly sampled 50 × third instar larvae. The surface of the larvae was sterilized by immersing the larvae in 75% ethanol for 90 s and then rinsing with sterilized ddH_2_O for three times. The larvae were dissected to remove the gut contents, which were homogenized with 1 mL sterilized ddH_2_O in a vortex mixer for 10 min to lyse the bacteria, before the DNA was extracted using a QIAamp®DNA Stool Mini Kit (QIAGEN, Gene Company Limited, China) following the manufacturer's protocol.

DNA of each of the bacteria-reared lines was analyzed using qPCR, by targeting the 16S-rRNA genes of *Enterococcus, Serratia*, and Enterobacteriaceae. Since the *Enterobacter* primers were difficult to design, we used the abundance of Enterobacteriaceae as a proxy to assess changes in abundance for *Enterobacter*. Primers are listed in Table S1. The general bacteria (Eub) primer set with total gut microbial DNA (Denman and Mcsweeney, 2006) was used to calibrate the relative abundance of the strains. Detailed methods for quantifying the bacteria are described in Xia et al. (2013). A Student's *t*-test was used to compare mean level of gut colonization by the three strains of bacteria.

### Effects of antibiotics on *P. xylostella* gut bacteria

Inhibition effects of antibiotics on the isolated gut bacteria were analyzed *in vitro* in MS medium (1 g yeast extract, 1.5 g K_2_HPO_4_, 0.5 g KH_2_PO_4_, 0.5 g (NH_4_)_2_SO_4_, 0.5 g NaCl, 0.2 g MgSO_4_, 0.05 g CaCl_2_, 0.02 g FeSO_4_, and 20 g agar in 1 L of ddH_2_O; pH 7.0) containing 1 mg mL^−1^ ciprofloxacin, 1 mg mL^−1^ levofloxacin, and 2 mg mL^−1^ metronidazole. The medium was spotted with 10 μL solution of each bacterium (*Enterobacter* sp. *Serratia* sp. and *Enterococcus* sp.; OD_600_ = 1.0) and the bacteria were cultured at 37°C for 24 h. In order to detect the efficiency of antibiotics against gut bacteria *in vivo*, cabbage leaves were dipped in a solution of 1 mg mL^−1^ ciprofloxacin, 1 mg mL^−1^ levofloxacin, and 2 mg mL^−1^ metronidazole in ddH_2_O for 10 min, air dried, and then put into Petri dishes. Control cabbage leaves were dipped in ddH_2_O. Next, 50 × first instar larvae were placed in a Petri dish and reared on the cabbage leaves until they reached the third instar. The cabbage leaves were renewed daily and there were three replicates of the treatments. The surface of the third instar larvae was sterilized by immersing in 75% ethanol for 90 s, before rinsing with sterilized ddH_2_O. The larvae were dissected to remove the gut contents and these were homogenized in 1 mL sterilized ddH_2_O. Next, 10 μL of the gut content suspension was used to culture bacteria in MS medium at 37°C for 24 h to assess the effect of the antibiotics on *P. xylostella* gut bacteria.

### Effects of vitamin C and acetylsalicylic acid

Vitamin C is an antioxidant that affects immune response (Hardie et al., 1991; Molina-Cruz et al., 2008; El-Gendy et al., 2010) and acetylsalicylic acid is believed to trigger eicosanoids (Claria and Serhan, 1995) that are an important component of immunity (Stanley and Miller, 2006). Previous studies have revealed that vitamin C repairs damage in *Drosophila melanogaster* caused by acephate (Rajak et al., 2017) and acetylsalicylic acid increases larval survival in *L. dispar* treated with Bt toxins (Broderick et al., 2010), so these two compounds that innately modulate immunity were selected to analyze their effect on the development of insecticide resistance in *P. xylostella*. Cabbage leaves were dipped in a suspension of vitamin C (5 mg ml^−1^), acetylsalicylic acid (10 mg ml^−1^) or ddH_2_O (as a control) for 10 min and air dried, before being added to Petri dishes, along with10 × first instar larvae. The leaves were renewed every day until the larvae had reached the third instar. There were three replicates of each treatment.

### Testing for resistance to chlorpyrifos in *P. xylostella*

Cabbage leaves were dipped in a solution of chlorpyrifos (50 g L^−1^) or in ddH_2_O (control) for 10 min, air dried and then put into Petri dishes, to which 10 × third instar larvae, which had been reared under the different bacteria, antibiotics, vitamin C and acetylsalicylic acid conditions, were added, after having been starved for 6 h. Survival was assessed at 24 h and 36 h, and there were three replicates of each treatment.

In order to study the effect of heat-killed bacteria on insecticide resistance, bacteria of the three strains (OD_600_ = 1.0) were heat-killed at 70°C for 15 min, and cabbage leaves were dipped in the suspensions for 10 min. Then, 10 × first instar larvae were added to Petri dishes, and reared on the cabbage leaves until the third instar. The third instar larvae were collected as for bioassay, as detailed above, and survival was assessed at 24 and 36 h; there were three replicates of each treatment. One-way ANOVA followed by an LSD *post-hoc* test was used to compare treatment means, using SPSS v. 23.

### Immune gene expression in *P. xylostella*

Batches of 10 × third instar larvae were randomly selected and starved for 6 h, before being transferred to Petri dishes and reared, for 24 h, on cabbage leaves treated with the different bacteria, antibiotic, vitamin C and acetylsalicylic acid conditions, as detailed above. Whole body total RNA of the larvae was extracted using qPCR following the methods described by Xia et al. (2015).

## Results

### Effect of gut bacteria on insecticide resistance

**Figure 1:**
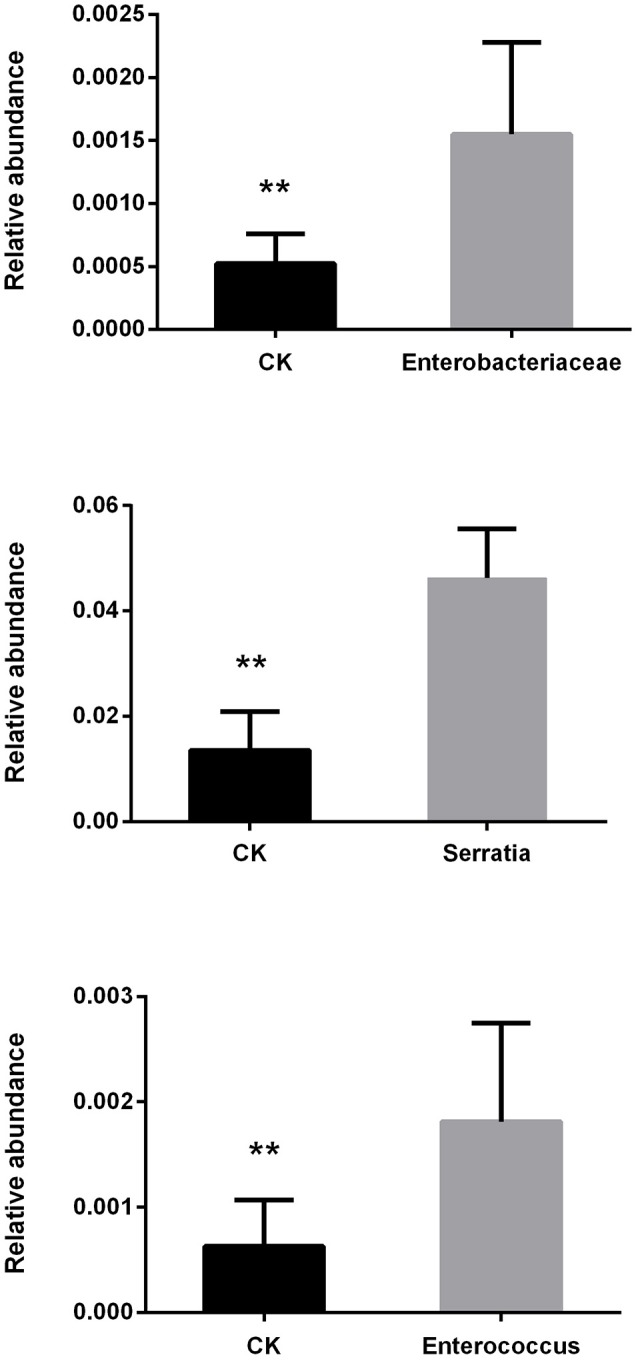
Changes in relative abundance of *P. xylostella* gut bacteria after consuming cabbage leaves inoculated with Enterobacteriaceae, *Serratia*, and *Enterococcus*. CK, control; error bars are standard deviation (SD), ^**^ indicates significant difference at *P* < 0.01.

The antibiotic treatment containing ciprofloxacin, levofloxacin, and metronidazole reduced bacterial growth *in vitro* (Figure S1) and similarly, gut bacteria collected from *P. xylostella* were inhibited by the antibiotics (Figure S2). We found that gut contents collected from *P. xylostella* that had been reared on a diet containing antibiotics contained no bacteria when cultured on antibiotic-free medium (Figure S3). When we examined the introduction of bacteria into the gut of *P. xylostella*, we found that all three strains of bacteria increased in abundance compared with the control (*Enterococcus* sp.: *t* = 3.43, *P* = 0.003; *Enterobacter* sp.: *t* = 4.00, *P* = 0.003; and, *Serratia* sp.: *t* = 8.24, *P* < 0.001; Figure 1).

**Figure 2:**
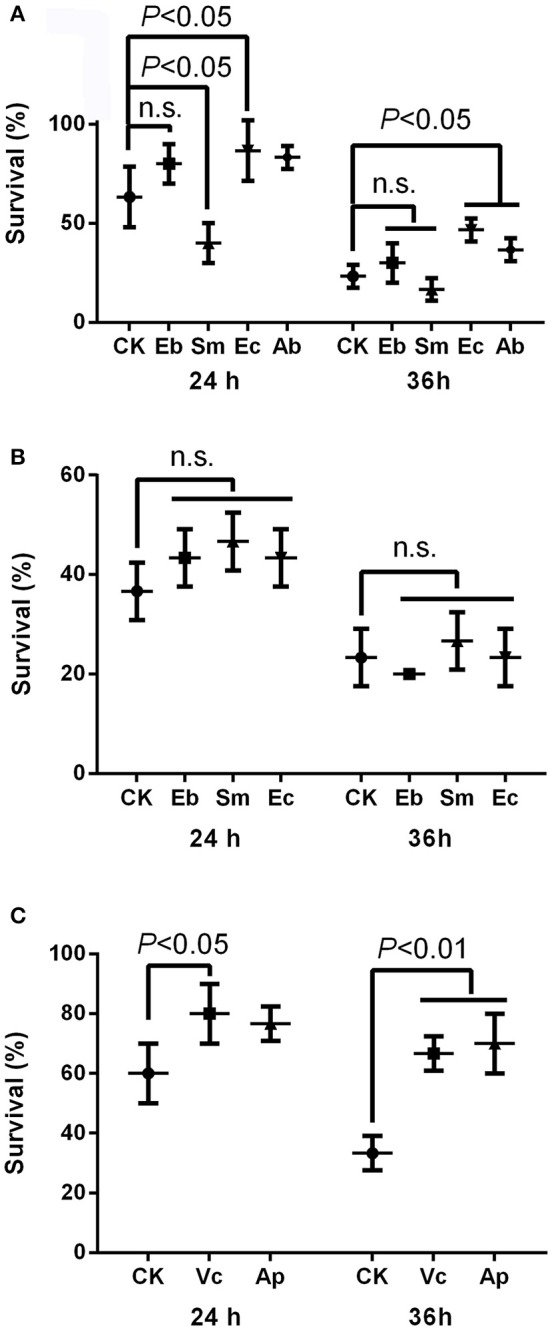
Effect of gut bacteria and compounds on mediation of insecticide resistance in *P. xylostella*. **(A)** Effects of live gut bacteria in mediation of resistance to chlorpyrifos; **(B)** effect of heat-killed gut bacteria in mediation of resistance to chlorpyrifos; and **(C)** effects of vitamin C and acetylsalicylic acid on mediation of resistance to chlorpyrifos. Eb, *Enterobacter* sp.; Sm, *Serratia* sp.; Ec, *Enterococcus* sp.; Ab, antibiotics; Vc, vitamin C, Ap, acetylsalicylic acid; and CK, control. Error bars are SD.

The insecticide resistance bioassay indicated that *Enterococcus* sp. significantly enhanced insecticide resistance in *P. xylostella* after 24 h [survival rate: 86.7 ± 15.3% compared to the 63.3 ± 15.3% in the control, *F*_(4, 10)_ = 8.02, *P* < 0.05] and 36 h [survival rate: 46.7 ± 5.8% compared to the 23.3 ± 5.8% in the control, *F*_(4, 10)_ = 8.71, *P* < 0.05], but *Serratia* sp. decreased insecticide resistance at 24 h [survival rate: 40.0% ± 10.0% compared to the 63.3 ± 15.3% in the control, *P* < 0.05], while *Enterobacter* sp. had no effect [survival rate: 80.0 ± 10.0% compared to the 63.3 ± 15.3% in the control, *P* = 0.07; Figure 2A]. Moreover, antibiotics enhanced the insecticide resistance significantly at 36 h [survival rate: 36.7 ± 5.8% compared to the 23.3 ± 5.8% in the control, *P* < 0.05; Figure 2A]. Heat-killed bacteria (Figure S4) had no effect on *P. xylostella* insecticide resistance, irrespective of strain, at 24 h [*F*_(3, 8)_ = 1.58, *P* = 0.268] or 36 h [*F*_(3, 8)_ = 0.90, *P* = 0.487] (Figure 2B).

### Effect of vitamin C and acetylsalicylic acid on insecticide resistance

The survival rate of *P. xylostella* was higher for larvae reared on vitamin C treated cabbage leaves (80.0 ± 10.0%) at 24 h than the control (60.0 ± 10.0%) [*F*_(2, 6)_ = 4.43, *P* < 0.05], and the survival rate of *P. xylostella* reared on cabbage leaves treated with acetylsalicylic acid (70.0 ± 10.0%) at 36 h was higher than the control (33.3 ± 5.8%) [*F*_(2, 6)_ = 22.20, *P* < 0.01; Figure 2C].

### Degradation of chlorpyrifos

**Figure 3:**
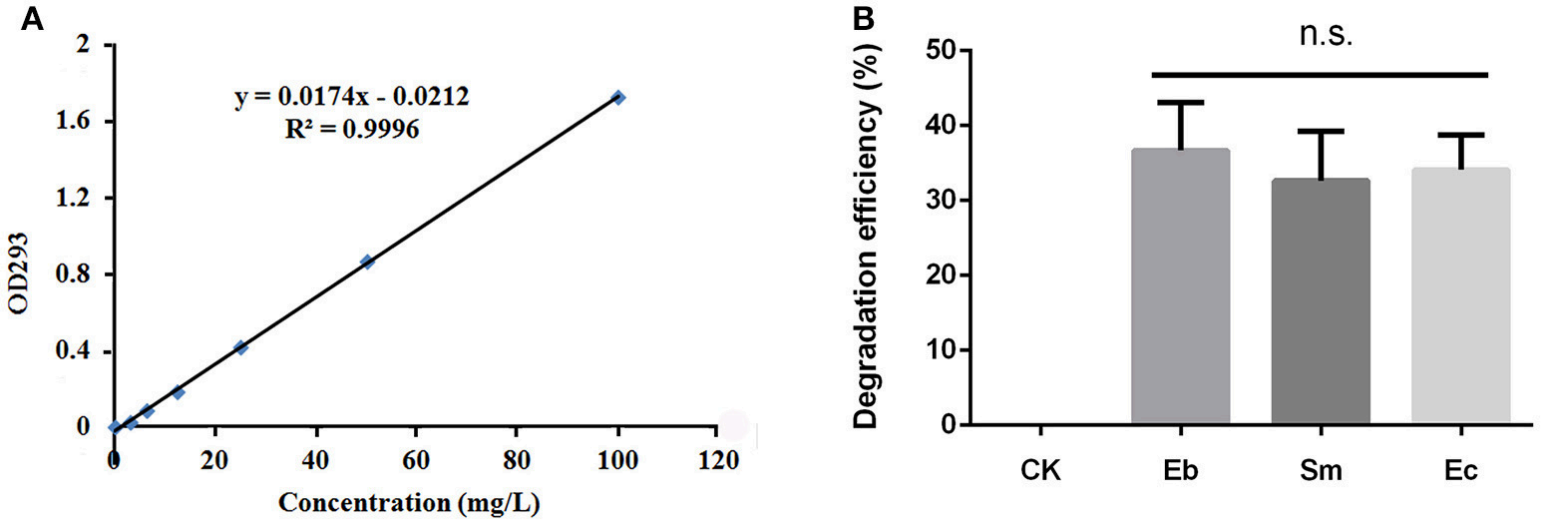
Degradation efficiency of *P. xylostella* gut bacteria on chlorpyrifos. **(A)** Standard curve of chlorpyrifos detected by ultraviolet spectrophotometry under OD_293_ nm; **(B)** degradation efficiency of chlorpyrifos by different gut bacteria. Eb, *Enterobacter* sp.; Sm, *Serratia* sp.; Ec, *Enterococcus* sp.; and CK, control. Error bars are SD; n.s. indicates no significant difference.

All three strains of bacteria degraded chlorpyrifos *in vitro*, with mean degradation efficiencies of 34.1 ± 4.6%, 36.7 ± 4.6%, and 33.0 ± 6.0% for *Enterococcus* sp., *Enterobacter* sp., and *Serratia* sp. respectively, but this difference was not significant overall [*F*_(2, 6)_ = 0.36, *P* = 0.711; Figure 3].

### Immune responses to gut bacteria and other compounds

**Figure 4:**
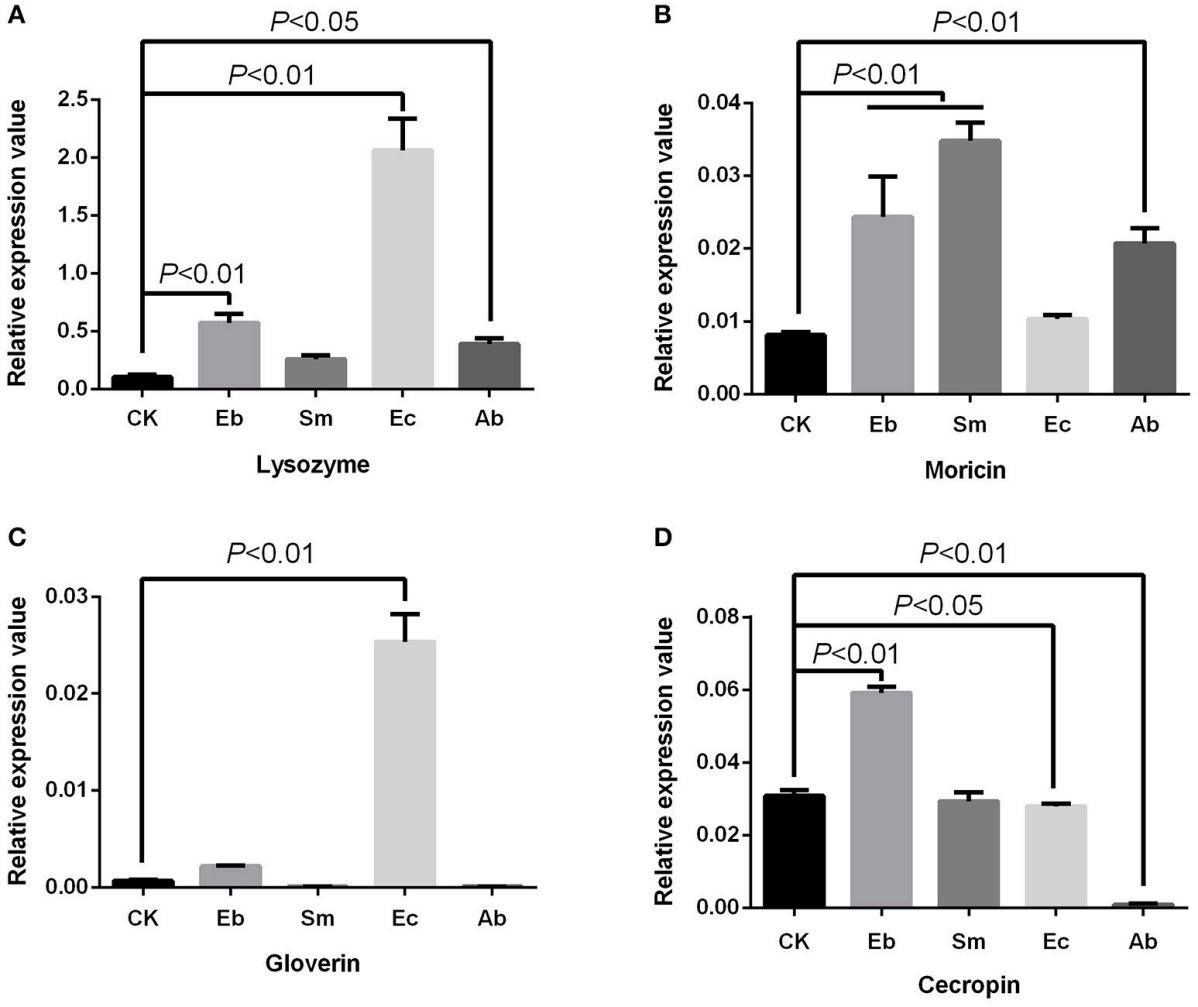
Effect of live gut bacteria on expression of AMPs in *P. xylostella*. **(A)** Expression of lysozyme; **(B)**, expression of moricin; **(C)** expression of gloverin; and **(D)** expression of cecropin. Eb, *Enterobacter* sp.; Sm, *Serratia* sp.; Ec, *Enterococcus* sp.; Ab, antibiotics; and CK, control. Error bars are SD.

We found that *Enterococcus* sp. induced the expression of gloverin [*F*_(4, 10)_ = 225.24, *P* < 0.01] and lysozyme [*F*_(4, 10)_ = 113.50, *P* < 0.01], but down-regulated expression of cecropin [*F*_(4, 10)_ = 560.12, *P* < 0.05] and had no effect on moricin expression [*F*_(4, 10)_ = 41.88, *P* = 0.377]. *Enterobacter* sp. induced cecropin [*P* < 0.01], moricin [*P* < 0.01], and lysozyme [*P* < 0.01] expression, but not that of gloverin [*P* = 0.184]. *Serratia* sp. induced moricin expression [*P* < 0.01] (Figure 4).

**Figure 5:**
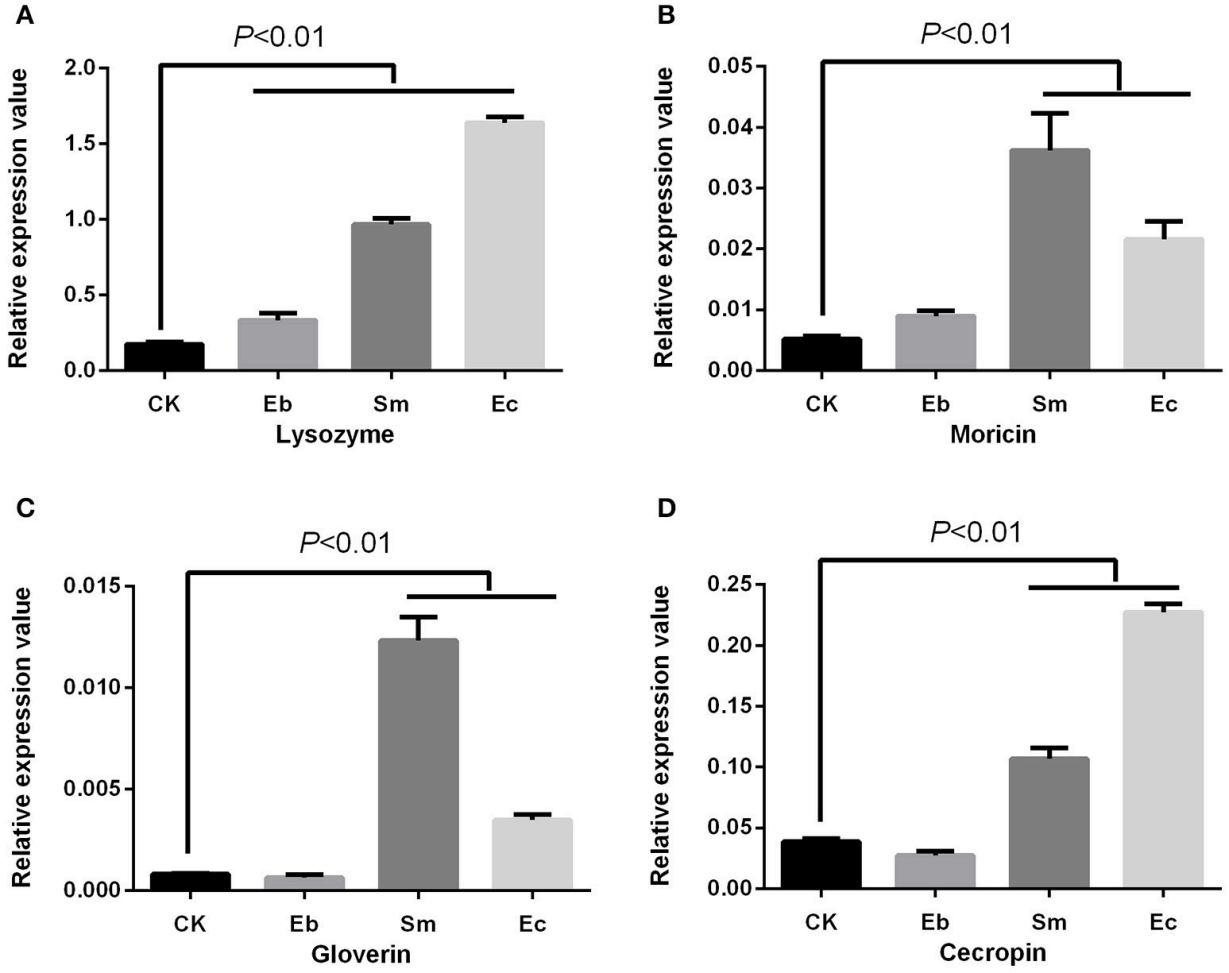
Effect of heat-killed gut bacteria on expression of AMPs in *P. xylostella*. **(A)** Expression of lysozyme; **(B)** expression of moricin; **(C)** expression of gloverin; and **(D)**: expression of cecropin. Eb, heat-killed *Enterobacter* sp.; Sm, heat-killed *Serratia* sp.; Ec, heat-killed *Enterococcus* sp.; and CK, control. Error bars are SD.

We studied the effect of heat-killed bacteria on expression of the *P. xylostella* immune genes, and found a different expression profile compared to the live cells. The heat-killed *Enterococcus* sp. and *Serratia* sp. induced the antimicrobial peptides (AMPs) of cecropin [*F*_(3, 8)_ = 680.05, *P* < 0.01], moricin [*F*_(3, 8)_ = 49.88, *P* < 0.01], gloverin [*F*_(3, 8)_ = 256.42, *P* < 0.01], and lysozyme [*F*_(3, 8)_ = 1005.25, *P* < 0.01]. Heat-killed *Enterobacter* sp. only induced lysozyme [*P* < 0.01] (Figure 5).

**Figure 6:**
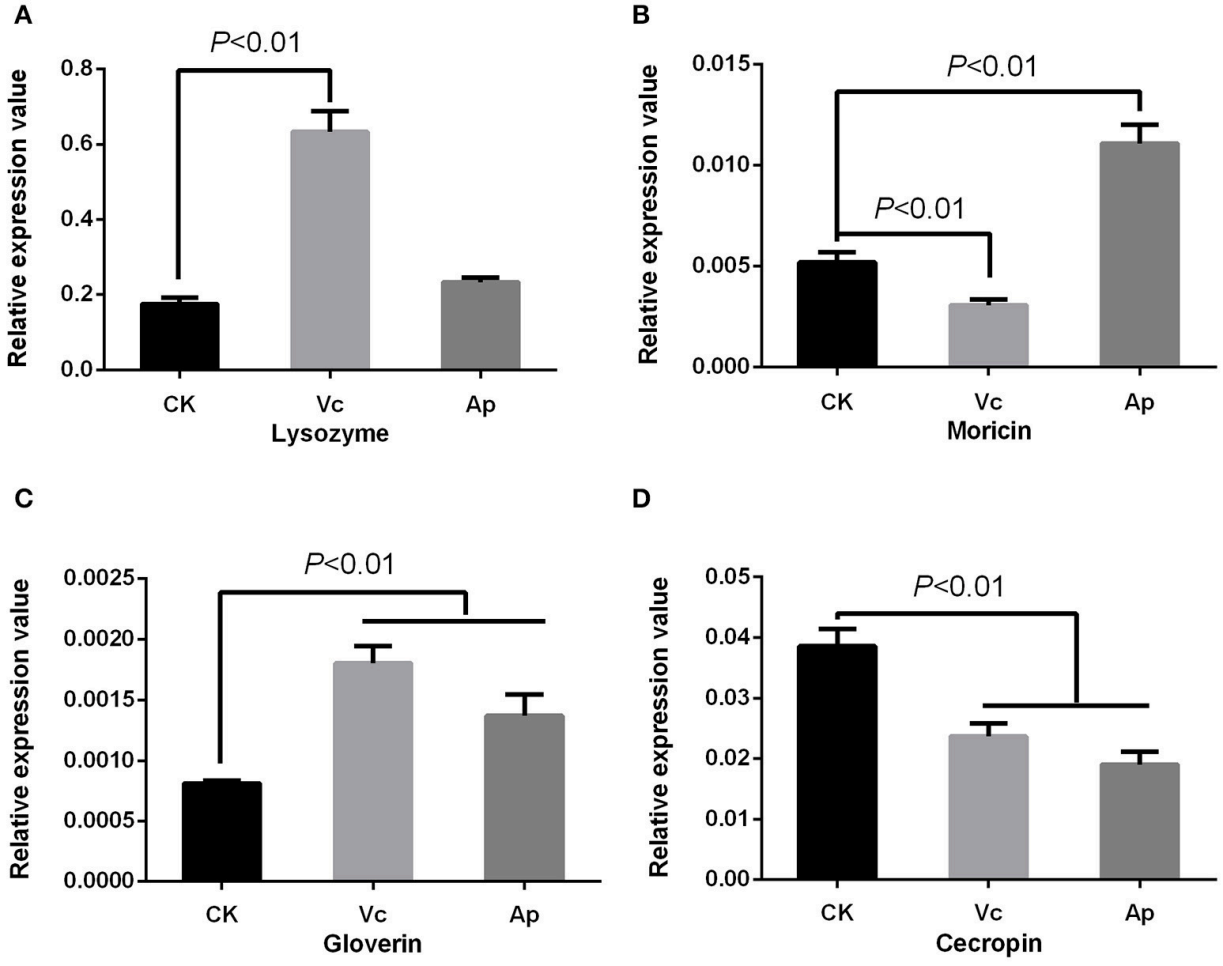
Effect of compounds on expression of AMPs in *P. xylostella*. **(A)** Expression of lysozyme; **(B)** expression of moricin; **(C)** expression of gloverin; and **(D)** expression of cecropin. Vc, vitamin C, Ap: acetylsalicylic acid; and CK, control. Error bars are SD.

Antibiotics were found to induce expression of moricin [*P* < 0.01] and lysozyme [*P* < 0.05], but down-regulate cecropin expression [*P* < 0.01] with no effect on gloverin expression [*P* = 0.573] (Figure 4). Vitamin C up-regulated the expression of gloverin [*F*_(2, 6)_ = 43.22, *P* < 0.01] and lysozyme [*F*_(2, 6)_ = 165.97, *P* < 0.01], but down-regulated cecropin [*F*_(2, 6)_ = 55.69, *P* < 0.01] and moricin [*F*_(2, 6)_ = 129.41, *P* < 0.01]. Finally, we found that acetylsalicylic acid up-regulated moricin [*P* < 0.01] and gloverin [*P* < 0.01], but down-regulated cecropin [*P* < 0.01] (Figure 6).

## Discussion

In our previous study, we reported an association between *P. xylostella* gut microbiota and insecticide resistance, especially for the phylum, Firmicutes (Xia et al., 2013). Here, we isolated three bacterial strains, *Enterococcus* sp. (Firmicutes), *Enterobacter* sp. (Proteobacteria), and *Serratia* sp. (Proteobacteria), from the guts of *P. xylostella* to evaluate their effect on and role in insecticide resistance. *Enterococcus* sp. was found to enhance resistance of *P. xylostella* to the insecticide chlorpyrifos while the Proteobacteria either had no effect (*Enterobacter* sp.) or decreased resistance (*Serratia* sp.). Moreover, we found that heat treatment to kill bacteria rendered all three species inactive. Our results suggest that gut microbiota may play an important role in *P. xylostella* insecticide resistance. Earlier research suggested that *L. dispar* larvae that were more susceptible to Bt toxin had a smaller population of gut bacteria, such as *Enterococcus faecalis*, leading to a decrease in pH of the midgut (Broderick et al., 2003, 2004). The mechanisms within the insect gut that affect resistance to Bt and chemical insecticides may differ, however, the composition of gut bacteria may affect host metabolism, immune system or degree of mutualism when the host is challenged with toxins.

The *in vitro* degradation of chlorpyrifos by *Enterococcus* was not significantly different from that of the other two strains (*Enterobacter* sp. and *Serratia* sp.), but its degree of improvement in resistance to chlorpyrifos was greatest. This indicates that there may be other mechanisms that are more important in gut bacteria mediated resistance to chlorpyrifos than direct metabolic degradation, especially given *Serratia* sp. enhanced the susceptibility of *P. xylostella* to chlorpyrifos. Our results differ from those reported by Kikuchi et al. (2012), who found that fenitrothion-degrading strains of *Burkholderia* mediated insecticide resistance in *Riptortus pedestris*, and by Cheng et al. (2017), who found that trichlorphon-degrading strains of *Citrobacter* sp. (CF-BD) contributed to the development of insecticide resistance in *B. dorsalis*. Their studies indicated that only strains with high pesticide degradation efficiency mediate insecticide resistance, since strains without degradation capacity cannot trigger host resistance to pesticides (Kikuchi et al., 2012; Cheng et al., 2017).

Immune responses are of obvious importance in insect pathogen interactions and our results suggest that immunological effects may be important in the development of resistance. The concept of gut bacteria influencing the development and response of host immunity in invertebrates and vertebrates is increasingly accepted (Kelly et al., 2005; Ryu et al., 2008; Hernández-Martínez et al., 2010), where modulation of host immune response by gut bacteria not only contributes to defense against pathogens (Dong et al., 2009), but also plays a role in insecticide resistance (Ericsson et al., 2009; Hernández-Martínez et al., 2010; Vezilier et al., 2013). Here, we found that *Enterococcus* sp., vitamin C, and acetylsalicylic acid enhanced resistance to chlorpyrifos in *P. xylostella* and also regulated the expression of *P. xylostella* AMPs. The expression of cecropin was down-regulated by these two compounds, while gloverin was up-regulated. The other bacteria, however, which did not confer insecticide resistance (either suppressing or having no significant effect) induced a different gene expression profile to that of *Enterococcus* sp. and the compounds treatments. Our previous transcriptome analysis of *P. xylostella* immune genes also revealed that the AMP genes of cecropin in chlorpyrifos resistant *P. xylostella* were down-regulated when compared with the susceptible strain (Xia et al., 2015). We speculate that pleiotropic effects of insect immunity and insecticide resistance may be a possible mechanism in gut bacteria mediated pesticide resistance in *P. xylostella*. For example, recent studies have shown pleiotropic effects of insect immunity on insecticide resistance in the *Anopheles* mosquito (Vontas et al., 2005, 2007), and Vezilier et al. (2013) reported that the AMP immune genes were increased in the insecticide-resistant mosquito *Culex pipiens* in isogenic lines. The mechanisms underlying these pleiotrophic effects remain unclear, although Rivero et al. (2010) proposed two possibilities: first, the effects of some genes that participate in immunity (from recognition to signal transduction) may be exploited in insecticide resistance and second, insecticide resistance and insect immunity may interact via trade-offs in resource allocation.

Insecticide resistance and immune response are energetically costly (Moret and Schmid-Hempel, 2000; Rivero et al., 2011). Insecticides damage the immune system of animals, such as decreasing immunocytes in birds exposed to chlorpyrifos (Singh et al., 2016), causing abnormal haemocyte counts in *Apis dorsata* (Perveen and Ahmad, 2017), stimulating cellular and humoral immunity in *Leptinotarsa decemlineata* (Coleoptera) and *Galleria mellonella* (Lepidoptera) (Dubovskiy et al., 2013), and affecting the nuclear factor-κB (NF-κB) that regulates expression of AMPs in *Apis mellifera* (Di et al., 2013). Gut symbioses are known to contribute to development of the insect gut immune system (Kelly et al., 2005; Ryu et al., 2010) and studies have suggested that acetylsalicylic acid modulates the immunity of the *L. dispar* and affect its susceptibility to Bt (Broderick et al., 2010) and vitamin C mitigates cytotoxicity of immunocytes in the *D. melanogaster* (Rajak et al., 2017). Thus, we propose that the gut bacteria, vitamin C and acetylsalicylic acid may prevent or restore damage to the *P. xylostella* immune system caused by insecticides, with this rather than bacterial degradation constituting the mechanism of the acquired resistance.

These results build on our earlier work that showed a causal link between the composition of the gut microbiota and chlorpyrifos resistance in *P. xylostella*. The mechanism mediating insecticide resistance of the gut bacteria remains unclear, however, Firmicutes and immunity appeared to be particularly important. Our studies suggest an interaction between gut microbiota and the insect immune system results in enhanced chemical insecticide resistance, but further studies are needed to support this hypothesis, and to clarify the other roles of the gut bacteria and chemicals used in this study. In conclusion, our study advances the understanding of the evolution of insecticide resistance in *P. xylostella*, and has identified areas of further research to fully elucidate the mechanisms in this species and more generally in other insect species with evolved insecticide resistance.

## Author contributions

XX and MY designed the project; XX, BS, and MX conducted the experiment; XX, MY, GG, and LV analyzed the data and wrote the paper.

### Conflict of interest statement

The authors declare that the research was conducted in the absence of any commercial or financial relationships that could be construed as a potential conflict of interest.

## Supplementary material

Figure S1 *P. xylostella* gut bacteria cultured on the plates containing antibiotics. Eb: *Enterobacter* sp., Sm: *Serratia* sp., Ec: *Enterococcus* sp., CK: the plates containing no antibiotics; Antibiotics: plates containing antibiotics with the concentration of 1 mg/mL Ciprofloxacin, 1 mg/mL Levofloxacin, 2 mg/mL Metronidazole.

Figure S2 *P. xylostella* gut content cultured on the plates containing antibiotics. CK: the plates containing no antibiotics; Antibiotics: plates containing antibiotics with the concentration of 1 mg/mL Ciprofloxacin, 1 mg/mL Levofloxacin, 2 mg/mL Metronidazole.

Figure S3 Gut contents cultured on the plates after the *P. xylostella* were reared with a diet (radish leaves) containing antibiotics. CK: *P. xylostella* reared with no antibiotics; Antibiotics: *P. xylostella* reared with a diet (radish leaves) containing with antibiotics at the concentration of 1 mg/mL Ciprofloxacin, 1 mg/mL Levofloxacin, 2 mg/mL Metronidazole.

Figure S4 Effect of heat on the *P. xylostella* gut bacteria. Eb: *Enterobacter* sp., Sm: *Serratia* sp., Ec: *Enterococcus* sp., CK: the control with no heat; Heat killed: the bacteria cultured under 70°C for 15 min.
