# Supplementary figures and images for: Gut Microbiota Mediate Insecticide Resistance in the Diamondback Moth, Plutella xylostella (L.)
Source: Front Microbiol. 2018 Jan 23;9:25. doi: 10.3389/fmicb.2018.00025 (PMC5787075; doi:10.3389/fmicb.2018.00025)

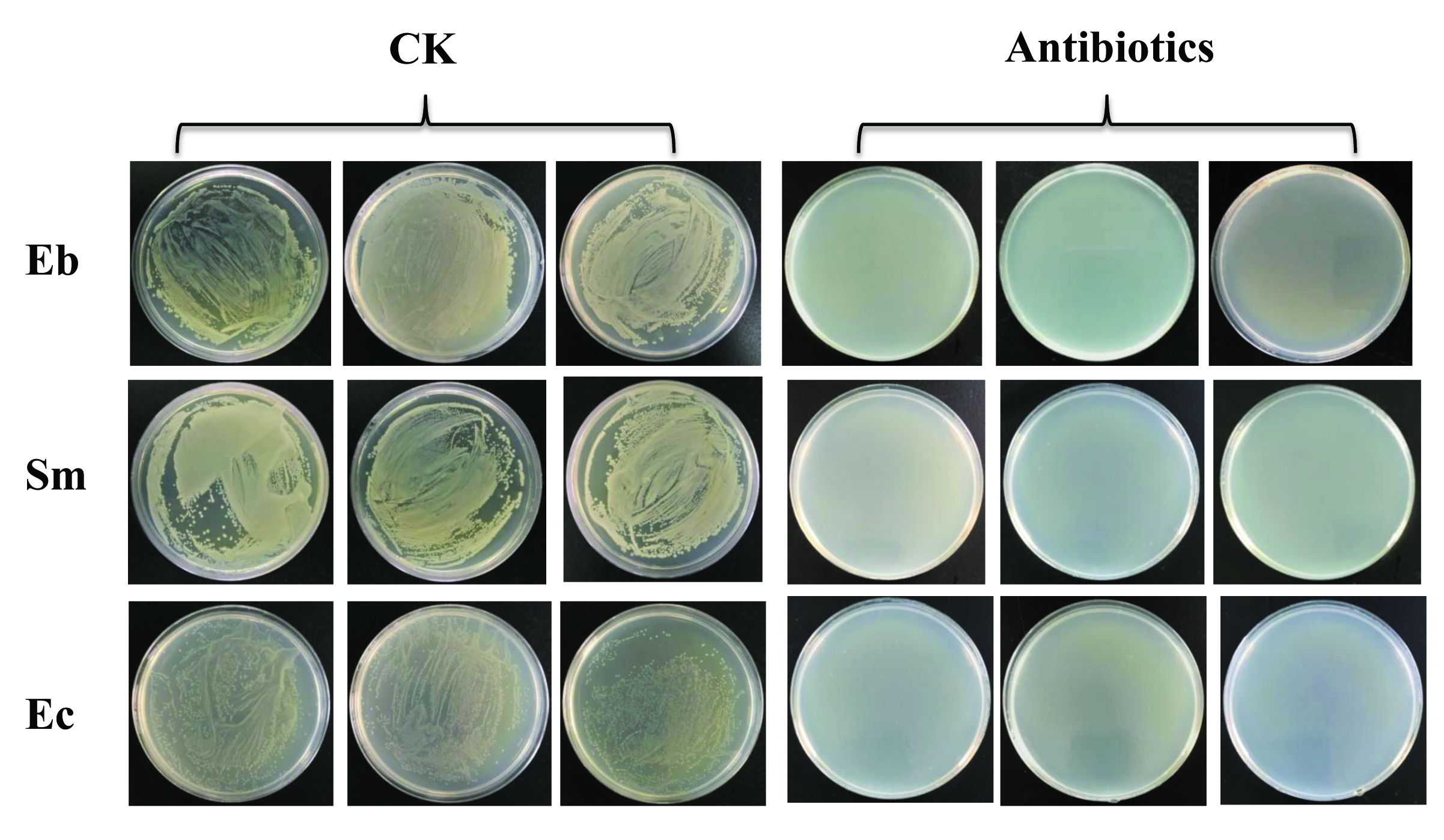

Supplement: Figure S1 — P. xylostella gut bacteria cultured on the plates containing antibiotics. Eb: Enterobacter sp., Sm: Serratia sp., Ec: Enterococcus sp., CK: the plates containing no antibiotics; Antibiotics: plates containing antibiotics with the concentration of 1 mg/mL Ciprofloxacin, 1 mg/mL Levofloxacin, 2 mg/mL Metronidazole. [file Image1.JPEG]

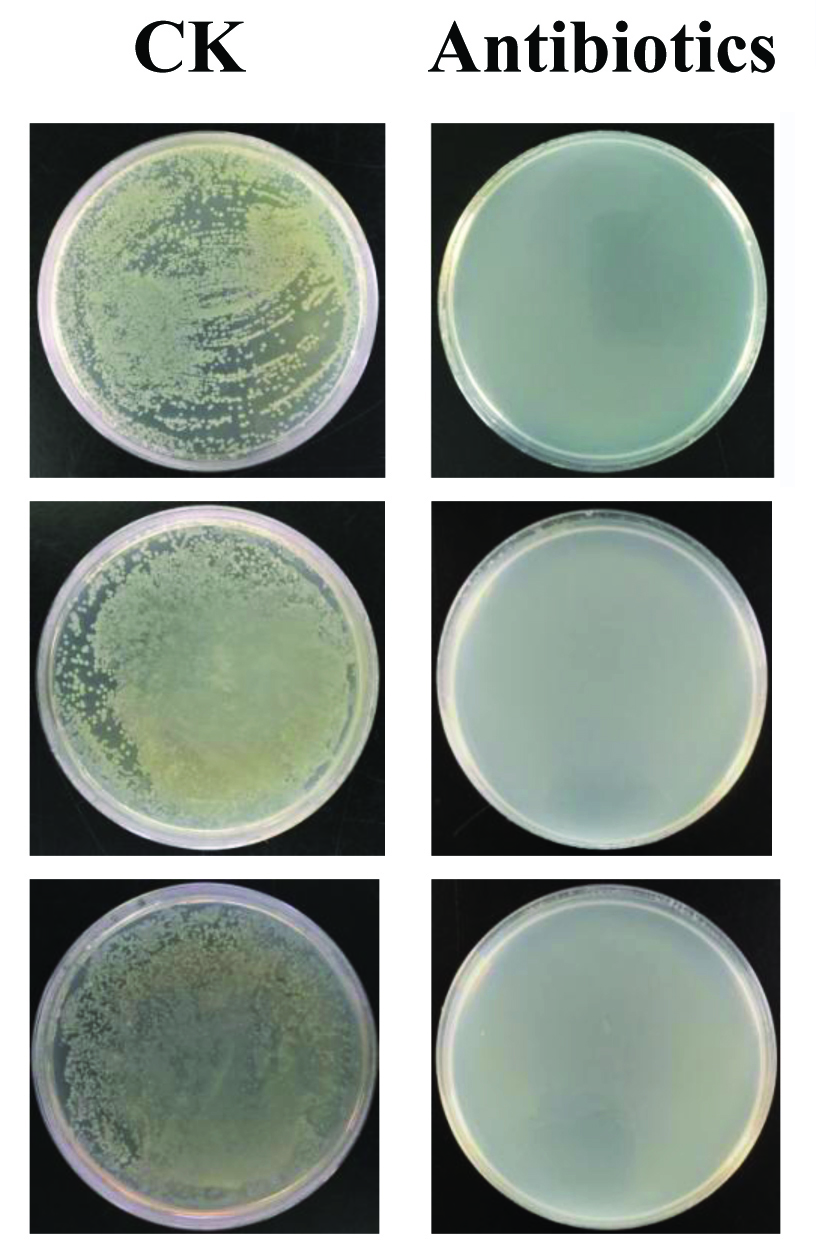

Supplement: Figure S2 — P. xylostella gut content cultured on the plates containing antibiotics. CK: the plates containing no antibiotics; Antibiotics: plates containing antibiotics with the concentration of 1 mg/mL Ciprofloxacin, 1 mg/mL Levofloxacin, 2 mg/mL Metronidazole. [file Image2.JPEG]

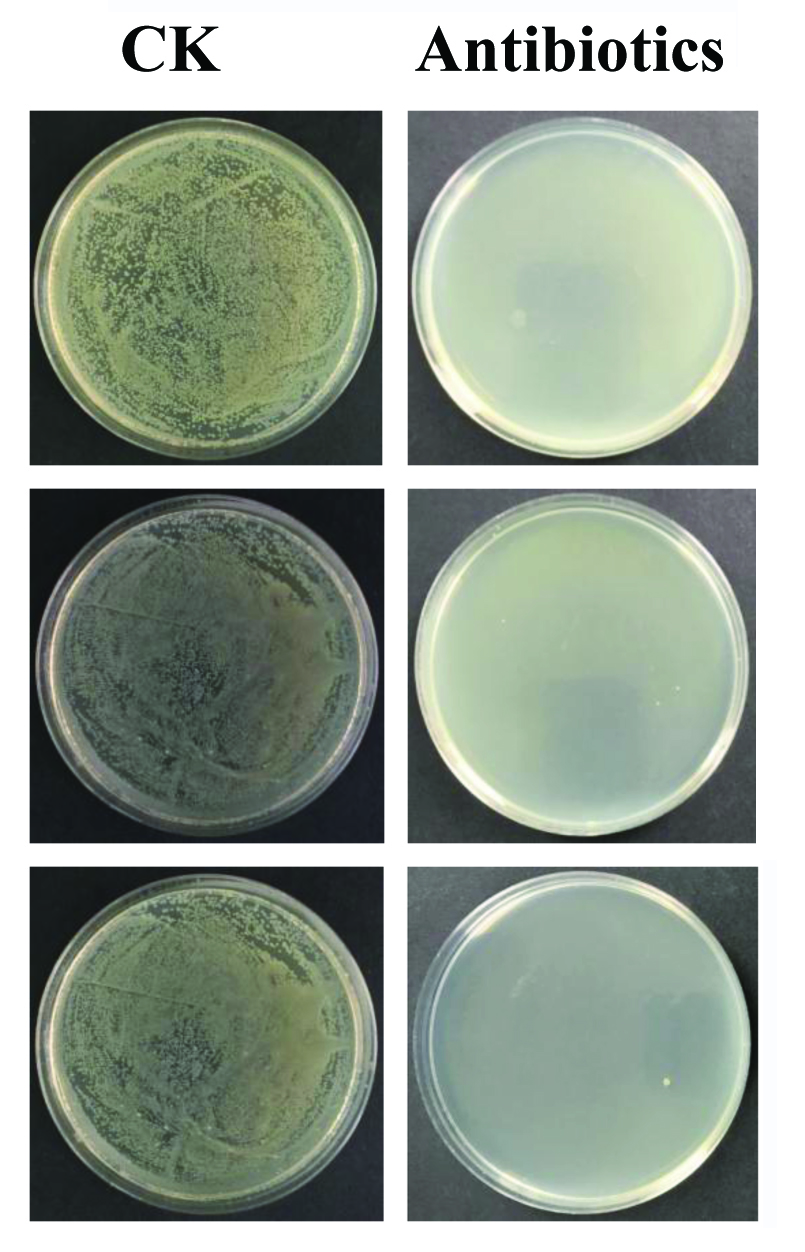

Supplement: Figure S3 — Gut contents cultured on the plates after the P. xylostella were reared with a diet (radish leaves) containing antibiotics. CK: P. xylostella reared with no antibiotics; Antibiotics: P. xylostella reared with a diet (radish leaves) containing with antibiotics at the concentration of 1 mg/mL Ciprofloxacin, 1 mg/mL Levofloxacin, 2 mg/mL Metronidazole. [file Image3.JPEG]

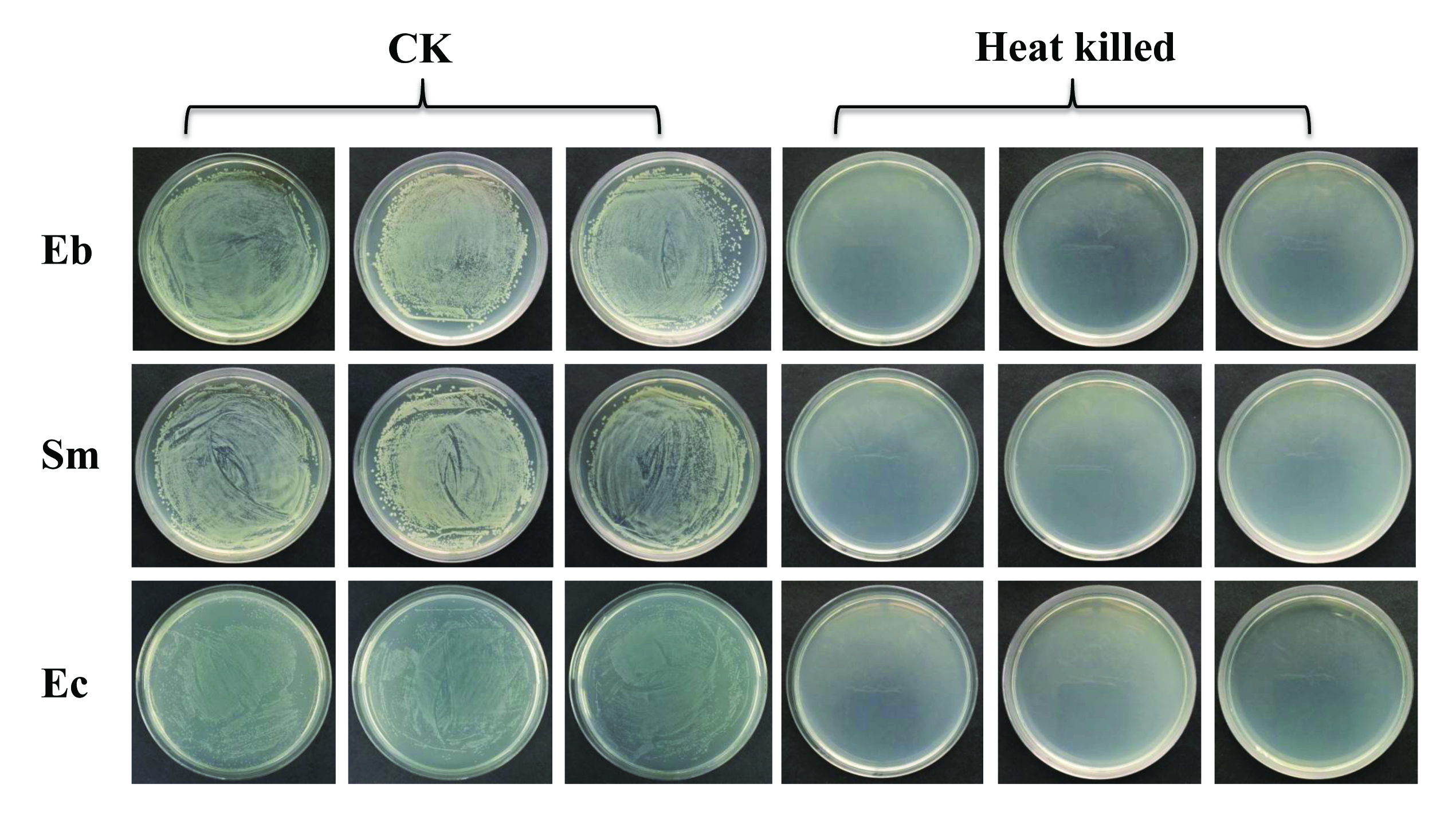

Supplement: Figure S4 — Effect of heat on the P. xylostella gut bacteria. Eb: Enterobacter sp., Sm: Serratia sp., Ec: Enterococcus sp., CK: the control with no heat; Heat killed: the bacteria cultured under 70°C for 15 min. [file Image4.JPEG]
