# Supplementary material for: Gut Microbiota Mediate Insecticide Resistance in the Diamondback Moth, Plutella xylostella (L.)
Source: Front Microbiol. 2018 Jan 23;9:25. doi: 10.3389/fmicb.2018.00025 (PMC5787075; doi:10.3389/fmicb.2018.00025)
Supplement: Supplementary file 5 [file Table1.DOC]

**Table S1 Primers used for the detection of gut bacteria**

| Gene name | Forward | Reverse | References |
| --- | --- | --- | --- |
| Eub | 5'- CGGCAACGAGCGCAACCC-3' | 5'-CCATTGTAGCACGTGTGTAGCC-3' | Denman and Mcsweeney, 2006 |
| Enterobacteriaceae | 5'-CATTGACGTIACCCGCGAGAAGAAGC-3' | 5'-CTCTACGAGCTCAAGCTTGC-3' | Bartosch et al., 2004 |
| *Enterococcus* | 5'-CCCTTATTGTTAGTTGCCATCATT-3' | 5'-ACTCGTTGTACTTCCCATTGT-3' | Rinttilä et al., 2004 |
| *Serratia* | 5'-GGTGAGCTTAATACGTTCATCAATTG-3' | 5'-GCAGTTCCCAGGTTGAGCC-3' | Iwaya et al., 2005 |

**References**

Bartosch, S., Fite, A., Macfarlane, G. T., and Mcmurdo, M. E. (2004). Characterization of bacterial communities in feces from healthy elderly volunteers and hospitalized elderly patients by using real-time PCR and effects of antibiotic treatment on the fecal microbiota. *Appl Environ Microbiol,* 70, 3575-3581.

Denman, S. E., and Mcsweeney, C. S. (2006). Development of a real-time PCR assay for monitoring anaerobic fungal and cellulolytic bacterial populations within the rumen. *FEMS Microbiology Ecology,* *58*, 572-582.

Iwaya, A., Nakagawa, S., Iwakura, N., Taneike, I., Kurihara, M., Kuwano, T., et al. (2005). Rapid and quantitative detection of blood *serratia marcescens*, by a real-time PCR assay: its clinical application and evaluation in a mouse infection model. *FEMS Microbiol Ecol,*248, 163-170.

Rinttilä, T., Kassinen, A., Malinen, E., Krogius, L., and Palva, A. (2004). Development of an extensive set of 16s rDNA-targeted primers for quantification of pathogenic and indigenous bacteria in faecal samples by real-time PCR. *J Appl Microbiol,* 97, 1166-1177.
